# Molecular changes in hypoxia-induced central neural circuits and nuclei

**DOI:** 10.4103/mgr.MEDGASRES-D-25-00148

**Published:** 2026-04-11

**Authors:** Xinyi Wang, Xiangyu Pan, Yu Zhou, Ziyang Jiang, Zhentong Qiu, Jiahui Ding, Shuangshuang Li, Wenxian Li, Yuan Han

**Affiliations:** Department of Anesthesiology, Eye & ENT Hospital of Fudan University, Shanghai, China

**Keywords:** cardiopulmonary functions, central nervous system, hypothalamic paraventricular nucleus, hypoxia, neural circuits, nucleus, oxygen, physiological homeostasis, rostral ventral lateral medulla, the nucleus of the solitary tract

## Abstract

**Facts**

1. Hypoxia activates a multi-level central regulatory network involving the carotid body and multiple brainstem nuclei that coordinate autonomic and cardiopulmonary responses.
2. Acute hypoxia induces rapid compensatory responses, while chronic hypoxia causes persistent central remodeling including neuronal injury and pathological hypertension.
3. Understanding molecular changes and neural circuit mechanisms provides a framework for treating hypoxia-related clinical diseases.

**Open Questions**

1. The dynamic interactions between different central nuclei and their coordination mechanisms during hypoxic responses remain incompletely understood.
2. The critical transition point from acute adaptive compensation to chronic pathological injury in hypoxia is not well defined.
3. The clinical translational value of potential therapeutic targets identified from central neural circuits requires systematic evaluation.

Hypoxia can be classified into two types based on its temporal characteristics: acute hypoxia and chronic hypoxia, posing a severe threat to the physiological homeostasis of the body. Hypoxia stimulates peripheral chemoreceptors and activates compensatory responses in the autonomic nervous system and cardiopulmonary functions. These responses rely on coordinated regulation by the carotid body and multiple nuclei in the central nervous system. Through specific neural pathways and molecular mechanisms, the body adapts to hypoxia and sustains survival. However, severe hypoxia may lead to irreversible damage or asphyxiation. In this review, we discuss recent research on central neural circuits and molecular changes in nuclei induced by hypoxia. It focuses on key regions associated with hypoxia, including the nucleus of the solitary tract, retrotrapezoid nucleus, rostral ventral lateral medulla, parabrachial nucleus, and hypothalamic paraventricular nucleus. From a neuroscience perspective, it elaborates on the effects of hypoxia on respiratory, cardiovascular, and other bodily functions. This understanding may help guide the treatment of hypoxia-related clinical diseases.

## Introduction

The central nervous system is extremely sensitive to hypoxia. Brain tissue consumes 23% of the total body oxygen, and cerebral blood flow accounts for 15% of cardiac output.^1^ Hypoxia leads to brain tissue damage through multiple pathways. These include neuronal injury, changes in neurotransmitters, disruption of neural circuits, and compensation mechanisms in brain function. These effects can cause acute neurological dysfunction and may result in long-term cognitive and motor impairments. Therefore, a thorough understanding of the nervous system’s response to hypoxia is essential for providing a solid theoretical basis for the treatment and prevention of related diseases.

Hypoxia is a pathological state characterized by insufficient oxygen supply or utilization at the tissue or cellular level. It can lead to energy metabolism disturbances and disrupt homeostasis. During acute hypoxic stress, the body employs multiple compensatory mechanisms to maintain the balance of oxygen supply and demand. Firstly, the carotid body is activated to enhance ventilation and improve oxygen uptake.^2^,^3^ Secondly, activation of the sympathoadrenal system increases cardiac output and redistributes blood flow,^4^ prioritizing the perfusion of vital organs, such as the heart and brain. Simultaneously, hypoxia-inducible factor-1α (HIF-1α) stabilizes through ubiquitin escape mechanism, and then regulates the transcription of target genes, such as erythropoietin and vascular endothelial growth factor.^4^,^5^ This process promotes erythropoiesis and angiogenesis to improve oxygen transport capacity. However, chronic or severe hypoxia can exceed compensatory thresholds. This leads to mitochondrial reactive oxygen species overproduction,^6^ adenosine triphosphate synthesis failure,^7^ and intracellular calcium homeostasis imbalance.^8^ In the central nervous system, this may cause neuronal apoptosis and cerebral edema. In the cardiovascular system, it can lead to reduced myocardial contractility and microcirculatory disturbances. This sequence may progress to multiple organ dysfunction syndrome.^4^,^9^

**Figure 1:**
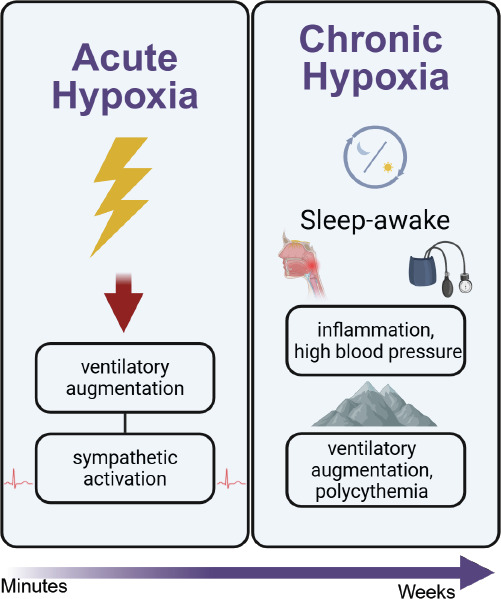
Temporal progression and physiological manifestations of hypoxic responses. Acute hypoxia (minutes) triggers immediate compensatory responses, including ventilatory augmentation and sympathetic activation, to restore oxygen homeostasis. Chronic hypoxia (weeks), particularly in the chronic intermittent hypoxia pattern characteristic of obstructive sleep apnea with repetitive sleep-awake cycles, induces sustained pathophysiological changes, including neuroinflammation, persistent hypertension, maintained ventilatory drive, and polycythemia as a systemic adaptation to prolonged oxygen deprivation. Created with BioRender.com.

Specifically, hypoxia can be classified into two types based on its temporal characteristics: acute hypoxia and chronic hypoxia (**Figure 1**). Acute hypoxia is commonly seen in situations such as respiratory paralysis, asphyxiation, acute poisoning, and acute massive hemorrhage. Acute hypoxia prompts the body to rapidly enter a defensive state to counteract its harmful effects. Chronic hypoxia can be further divided into chronic sustained hypoxia (CSH) and chronic intermittent hypoxia (CIH). CSH is typically associated with chronic lung disease, heart disease, and high-altitude environments. It represents a continuous pathological state. CIH is the fundamental pathophysiological change in obstructive sleep apnea (OSA). Patients with OSA experience recurrent obstructive respiratory events that lead to cycles of hypoxia and reoxygenation. This condition results in increased sympathetic nervous activity and pathological hypertension. Therefore, OSA patients are often in a state of CIH. Currently, the CIH model is widely used to study autonomic nervous system changes and respiratory variations observed in OSA.^10^,^11^

Currently, clinical attention to the relationship between disease and brain oxygen levels is increasing. With advancements in magnetic resonance imaging, it is widely used for non-invasive studies of brain anatomy and vascular responses. Mapping the clinical oxygen extraction fraction using magnetic resonance imaging is crucial for non-invasive characterization and monitoring of brain oxygen metabolism and cerebrovascular reserve in patients with hypoxia-related disorders.^12^ This approach offers substantial advantages in detecting relative physiological changes between normoxia and hypoxia. Functional magnetic resonance imaging allows measurement of changes in cerebral blood flow and blood oxygen level-dependent signals during hypoxia.^13^,^14^ For example, hypoxia can lead to cognitive impairments.^15^ Functional magnetic resonance imaging enables the analysis of regional differences in brain oxygen metabolism during hypoxia and their association with cognitive deficits,^16^ providing insights into the physiological mechanisms underlying hypoxia-induced cognitive dysfunction. In basic research, optogenetics permits precise control of central hypoxia-related neurons.^17^ Combined with respiratory plethysmography and pulse oximetry, it allows real-time monitoring of respiration patterns, heart rate, and blood oxygen in mice.^17^,^18^ This provides a solid foundation for studying the effects of hypoxia-related diseases on cardiopulmonary reflex.

This review aims to systematically synthesize current knowledge on the central neural mechanisms underlying hypoxia-induced cardiorespiratory and autonomic regulation, with emphasis on key brainstem and hypothalamic nuclei, including the nucleus of the solitary tract (NTS), retrotrapezoid nucleus (RTN), rostral ventral lateral medulla (RVLM), parabrachial nucleus (PB), and paraventricular nucleus (PVN). We also compare the differential responses and molecular adaptations between acute and chronic hypoxia exposure to identify potential therapeutic targets for hypoxia-related disorders, such as OSA and high-altitude illnesses.

## Search Strategy

Relevant literature was retrieved through an electronic search of PubMed databases from 2014 to May 20, 2025. The search strategy and selection criteria used the following keywords: hypoxia, central nervous system, nucleus of the solitary tract, retrotrapezoid nucleus, rostral ventral lateral medulla, parabrachial nucleus, paraventricular nucleus, neural circuits, chemoreceptors, carotid body, respiratory control, cardiovascular regulation, chronic intermittent hypoxia, obstructive sleep apnea. We used various combinations of the above search terms to comprehensively access the literature. Initially, we reviewed the relevance of the title and abstract to our target content. If they were considered relevant, then the whole paper was accessed to ensure there were suitable descriptions of central hypoxic mechanisms and neural circuit regulation. Articles published in English and focusing on central neural responses to hypoxia or cardiorespiratory regulation were included. Articles that did not focus on central hypoxic mechanisms or neural circuit regulation were excluded, as were those not published in English.

## The Nucleus of the Solitary Tract and Hypoxia

The NTS is the first integration point for visceral sensory inputs. This area receives input from baroreceptors and peripheral chemoreceptors, including the carotid body, as well as visceral inputs from the gastrointestinal tract.^19^,^20^,^21^,^22^,^23^ It serves as a key brainstem hub for coordinating sympathetic activity and maintaining cardiovascular homeostasis. Notably, the functional integration of NTS relies not only on peripheral inputs but also on descending modulation from various nuclei in the forebrain and brainstem, including structures in the lateral hypothalamus, paraventricular hypothalamus, and central amygdala within the limbic system.^24^,^25^,^26^ These multi-layered neural regulations shape the excitability characteristics of NTS neurons, and ultimately produce comprehensive responses to chemical afferents. Finally, these responses will contribute to metabolic homeostasis and affect cardiopulmonary function^23^ as well as food intake and digestion.^27^ NTS contains three functionally specialized neuron groups: glutamatergic, catecholaminergic (mainly noradrenergic), and γ-aminobutyric acid (GABA)ergic neurons. It dynamically regulates autonomic function through their neurotransmitter release and synaptic plasticity mechanisms.^28^,^29^ It sends widespread projections to central nervous system regions, including pre-Bötzinger complex,^30^ Bötzinger complex,^31^ lateral respiratory group in the medulla oblongata,^32^ caudal and rostral ventrolateral medulla,^33^ RTN,^34^ locus coeruleus,^35^ PB,^36^ periaqueductal gray matter,^36^ among others. This multidimensional output enables the NTS to coordinate the stability of multiple physiological functions.

### Role of the nucleus of the solitary tract in acute hypoxia

#### Nucleus of the solitary tract: the primary relay station that mediates peripheral acute hypoxic signals to the central nervous system

**Figure 2:**
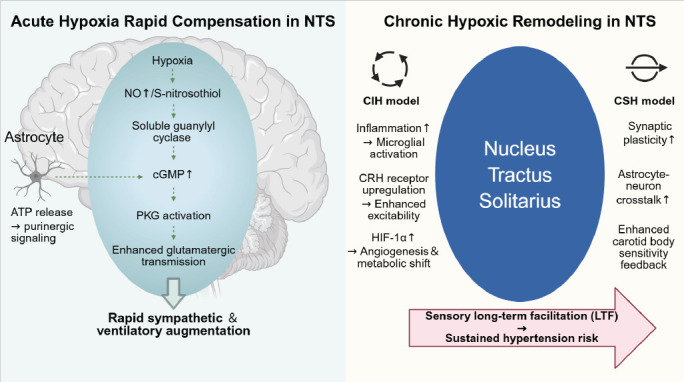
Differential mechanisms of acute *versus* chronic hypoxia-induced plasticity in the NTS. Acute hypoxia triggers rapid compensatory responses through NO/cGMP/PKG signaling and ATP-mediated purinergic pathways, enhancing glutamatergic transmission to drive immediate sympathetic and ventilatory augmentation. Chronic hypoxia induces maladaptive plasticity that differs by pattern: CIH involves neuroinflammation, CRH receptor upregulation, and HIF-1α-mediated metabolic reprogramming; CSH involves synaptic plasticity, enhanced astrocyte-neuron crosstalk, and augmented carotid body feedback. Both patterns result in sensory long-term facilitation and sustained hypertension risk. Mechanisms shown separately for clarity; substantial overlap exists between CIH and CSH pathways, with HIF-1α, neuroinflammation, and synaptic remodeling being common features of chronic hypoxia. Created with BioRender.com. ATP: Adenosine triphosphate; cGMP: cyclic guanosine monophosphate; CIH: chronic intermittent hypoxia; CRH: corticotropin-releasing hormone; CSH: chronic sustained hypoxia; HIF-1α: hypoxia-inducible factor-1 alpha; LTF: long-term facilitation; NO: nitric oxide; NTS: the nucleus of the tractus solitarius; PKG: protein kinase G.

During acute hypoxia, the NTS serves as a relay station mediating communication between peripheral and central systems (**Figure 2**). Research has found that the NTS is closely associated with the sigh reflex induced by acute hypoxia.^17^ In acute hypoxia, the carotid body senses the reduced partial pressure of oxygen in arterial blood and relays a stimulus to the NTS via the glossopharyngeal nerve. Then, gastrin-releasing peptide positive neuronal populations in the NTS are activated. These neurons project to glutamatergic neurons in pre-Bötzinger complex to induce a sigh-like response. This process is critical for reversing alveolar collapse (atelectasis) and maintaining normal lung function. Previous research has also indicated that increased diaphragm activity during acute hypoxia depends on elevated glutamate levels in the NTS.^37^,^38^ Therefore, the NTS is a key nucleus for maintaining alveolar reopening and enhancing oxygenation under conditions of acute hypoxia.

#### Ventilatory compensation mediated by redox regulation in the nucleus of the solitary tract

The central hypoxic ventilatory compensatory mechanism is a key neural pathway for maintaining oxygen homeostasis. Research indicates that the nitric oxide synthase system, densely distributed in the NTS, plays a key role in this process. When the body is exposed to hypoxic stimulation, the nitric oxide synthase in the NTS is specifically activated. And this activation produces S-nitrosothiol,^39^ the S-nitrosothiol then activates the soluble guanylate cyclase-cyclic guanosine monophosphate pathway within 30 seconds, and leads to compensatory increases in minute ventilation to counteract the damage caused by hypoxia.^40^ This rapid response mechanism not only alleviates the damage caused by hypoxia to tissues, but also improves blood oxygen supply in the brainstem through inducing local vasodilation, thus forming a positive feedback loop for “oxygen supply-consumption” dynamic balance.^41^ Notably, this regulation exhibits significant redox sensitivity. Experiments have confirmed that external antioxidants can completely block this compensatory process.^42^ This indicates that its activity is dynamically regulated by the body’s redox state. Therefore, the nitric oxide synthase/S-nitrosothiol-based rapid response mechanism reveals the precise neural regulation in coping with hypoxic challenges and also provides new molecular targets for clinical interventions in respiratory compensation disorders.

#### Circuit regulation of the cardiopulmonary reflex

The NTS is crucial for shaping sympathetic and respiratory responses to hypoxia.^25^ It coordinates adaptive changes in sympathetic nervous excitability and respiratory rhythms through a complex neural regulatory network. Recent research further reveals that the projection from PVN to NTS can significantly enhance NTS neuronal activation and is necessary for full cardiorespiratory responses to hypoxia. However, when the pathway was inhibited, the hypoxic ventilatory response (HVR) in mice was weakened.^43^ Thus, the NTS serves not only as the central hub for processing hypoxic signals but also as the key “decision center” for initiating cardiopulmonary compensatory responses.

### Role of the nucleus of the solitary tract in chronic hypoxia

#### Remodeling of the nucleus of the solitary tract internal microenvironment

Chronic hypoxia influences the physiological function of the NTS through neuroplasticity and inflammation regulation (**Figure 2**). CIH reshapes the microenvironment of the NTS through two pathways. First, chronic hypoxia promotes the formation of an inflammatory microenvironment. CIH increases the mRNA expression levels of pro-inflammatory cytokines, such as interleukin-1 beta, interleukin-6 and tumor necrosis factor-alpha in the caudal NTS,^44^ driving a local neuroinflammatory cascade. Second, chronic hypoxia induces an abnormal increase in corticotropin-releasing hormone (CRH) receptor 2 in the NTS. This activation stimulates the sympathetic-adrenal axis and promotes the development of hypertension.^44^ Notably, CIH has a bidirectional effect on the cardiovascular system. Short-term exposure can enhance myocardial protection through heat shock protein-mediated antioxidant pathways. However, prolonged stimulation triggers excessive inflammatory responses that lead to endothelial dysfunction, ultimately exacerbating tissue damage.^45^,^46^ These findings clarify the molecular pathways by which CIH regulates NTS function through neuroinflammation and synaptic plasticity. They also reveal the coexisting pathological features of “damage-compensation.”

#### Synaptic plasticity and neural circuit reconfiguration of the nucleus of the solitary tract

As the central hub of hypoxic stress regulation, changes in the activity of glutamatergic neurons within the NTS are crucial for the body’s adaptation and response to chronic hypoxia. Research has shown that CIH can induce long-term synaptic depression in the NTS by reducing the number of functional synapses. However, this process does not alter the vesicular release or quantal size of individual synapses.^46^ Notably, this synaptic depression does not impair the overall functional activity of the NTS. After chronic hypoxia exposure, synaptic activity and c-Fos expression levels in NTS neurons exhibit a compensatory increase.^46^,^47^,^48^ At the molecular level, the plasticity of different glutamate receptors uniquely influences respiratory reflexes under hypoxia.^38^,^49^ CSH enhances the HVR by promoting phosphorylation modifications of α-amino-3-hydroxy-5-methyl-4-isoxazolepropionic acid receptors, thereby increasing their channel open probability.^37^ And the key signaling molecule HIF-1α plays a pivotal regulatory role in this process. Specific knockout of HIF-1α in glutamatergic neurons of the NTS significantly blocks the ventilatory adaptation of mice to CSH, while the basic respiratory rhythm under normoxic conditions remains unaffected.^50^ Furthermore, the central nervous system prevents excessive compensation through dynamic balance mechanisms. CSH enhances the norepinephrine-mediated α2-adrenergic receptor’s inhibitory effect on glutamate release from the NTS, effectively modulating neuronal excitability thresholds.^51^ At the same time, neural projections from NTS to PVN and synaptic integration within the PVN are also altered^52^; these changes result in the enhanced PVN neuronal activity.^53^ Thus, these findings elucidate how the NTS promotes compensatory responses during chronic hypoxia through mechanisms such as synaptic plasticity, dynamic receptor regulation and neural circuit remodeling.

#### Glia-neuron interaction in the nucleus of the solitary tract

**Figure 3:**
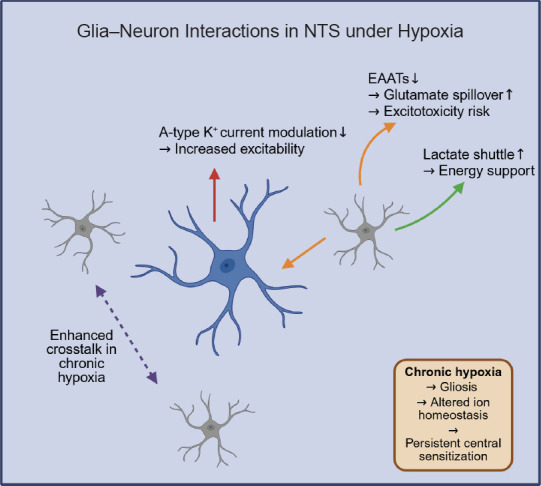
Glia–neuron interactions in the NTS under hypoxia. Chronic hypoxia activates NTS astrocytes, which modulate neuronal excitability through reduced A-type K^+^ currents, impaired glutamate reuptake via downregulated EAATs, and enhanced metabolic support via lactate shuttling. This bidirectional crosstalk contributes to gliosis, altered ion homeostasis, and persistent central sensitization underlying cardiorespiratory dysfunction. Created with BioRender.com. EAATs: Excitatory amino acid transporters; NTS: the nucleus of the solitary tract.

As a key regulator of neuron-glial interactions, astrocytes in the NTS dynamically modulate the synaptic microenvironment and ionic homeostasis (**Figure 3**). During chronic hypoxia, chronic hypoxia can reshape the functional coupling patterns of astrocytes and excitatory neurons in the NTS. This, in turn, affects the integration and amplification of peripheral chemoreflex signals in the central nervous system.^54^ Mechanistic studies reveal that astrocytes are activated first during chronic hypoxia.^55^ This activated state regulates synaptic transmission efficiency through dual pathways. First, during chronic hypoxia stimulation, the inhibitory effect of astrocytes on the A-type potassium current in NTS neurons decreases. This reduction lowers the excitability threshold of the neurons and increases the amplitude of postsynaptic currents triggered by incoming fiber stimulation.^56^ Second, astrocytes in the NTS significantly amplify the strength of chemosensory signals by enhancing α-amino-3-hydroxy-5-methyl-4-isoxazolepropionic acid or N-methyl-D-aspartate receptor-mediated synaptic currents. This increases the sensitivity of CSH rats to cardio-pulmonary responses induced by pressure and chemoreflex stimuli.^57^ Additionally, excitatory amino acid transporters (EAATs) in the membrane of NTS astrocytes act as key participants in regulating extracellular glutamate. In rats exposed to short-term sustained hypoxia, EAATs play a bidirectional regulatory role in processing chemoreflex incoming information. Short-term exposure upregulates EAAT2 expression, and accelerates glutamate reuptake in the synaptic cleft to maintain neurotransmitter balance. In contrast, long-term exposure leads to EAAT functional exhaustion, resulting in extracellular glutamate accumulation and exacerbating synaptic hyperexcitability.^58^,^59^,^60^ Moreover, chemogenetic modulation of astrocytes indicates that when NTS astrocyte activity is inhibited, the sympathetic nerve overactivity and hypertension induced by CIH can be reversed. This provides direct evidence for reversing hypoxic cardiovascular damage.^61^ Thus, these findings highlight the clinical potential of NTS glia-neuron interaction in chronic hypoxia-related cardiopulmonary diseases.

### Role of the nucleus of the solitary tract in endocrine changes induced by hypoxia

As an integrative center for multisystem regulation, NTS not only coordinates cardiopulmonary compensatory reflexes under hypoxic stress but also maintains a dynamic balance of metabolism and internal environmental homeostasis through precise neuroendocrine pathways. Research has found that activation of the leptin signaling pathway in the caudal NTS significantly enhances brain glucose homeostasis regulation induced by hypoxia. Hypoxic exposure upregulates the expression of leptin receptor. This upregulation promotes carotid body chemoreceptor-mediated brain glucose retention, and this process occurs independently of changes in the electrical potential due to persistent neuronal activation.^62^ Furthermore, the dynamic interactions of neuropeptides within the NTS are critical for hypoxic adaptation. Oxytocin (OT) and CRH enhance the overall regulatory capacity against hypoxic stress by synergistically activating NTS neuronal cluster firing.^63^ To sum up, these findings highlight the central role of the NTS in regulating metabolic-neuroendocrine interactions.

### Role of the nucleus of the solitary tract in hypoxic responses under pathological conditions

In pathological states, NTS neurons also show altered responses to hypoxia. For example, in the Parkinson’s disease model (6-hydroxydopamine), the number of paired-like homeobox 2b (Phox2b) positive glutamatergic neurons in the NTS will decrease. This reduction leads to impaired acute hypoxic respiratory response.^64^ Thus, the regulatory role of the NTS in hypoxia persists in pathological conditions. However, the specific mechanisms of this regulation may differ from those in physiological states and require further exploration.

NTS coordinates the body’s compensatory response to hypoxic stress through a triadic regulatory model of rapid signaling-metabolic integration-plasticity adaptation. Previous studies have shown that its functional network encompasses multidimensional interactions among the nervous, glial, immune, and endocrine systems, providing a new perspective for investigating mechanisms and therapeutic strategies for respiratory-metabolic diseases.^65^,^66^ Future research should focus on cell subtype-specific circuits, epigenetic dynamics, and pathological transformation thresholds. This focus will promote the application of precision medicine in hypoxia-related diseases.

## Retrotrapezoid Nucleus and Hypoxia

The RTN is an important central chemoreceptor region located in the ventrolateral medulla, near the ventral part of the facial nerve. There are significant differences in this nucleus across species. The RTN region in rats contains approximately 2000 chemosensitive neurons, while in mice, this number decreases to about 800.^67^ From the neurochemical perspective, RTN neurons specifically express Phox2b, neurokinin-1 receptors, vesicular glutamate transporter 2 (VGlut2), TWIK-related acid-sensitive K^+^ channel 2 channels, G protein-coupled receptor 4, and galanin, while lacking the expression of GABA, glycine, acetylcholine or catecholamines.^68^,^69^ This unique combination of molecular markers distinguishes RTN from adjacent nuclei and establishes a specific neurotransmitter regulatory network. RTN neuronal projections are highly selective, with their glutamatergic output mainly targeting four key respiratory regulatory centers: the ventral respiratory column, the Köliker-Fuse nucleus, the lateral PB (LPB), and NTS.^70^,^71^ RTN receives regulatory input from multiple brain regions. Notably, cholinergic inputs from the pedunculopontine tegmental nucleus and the preinspiratory complex can enhance chemoreceptor activity.^34^ Additionally, serotonergic inputs from the caudal and dorsal raphe nuclei can increase the RTN’s sensitivity to carbon dioxide (CO_2_),^72^ while inputs from the spinal cord to the insular cortex may contribute to higher-level respiratory regulation.^73^

In the complex respiratory control network, RTN neurons play a significant role in sensing changes in CO_2_ levels. First, optogenetic studies have shown that activating RTN neurons can significantly enhance ventilatory efficiency, evidenced by an increased respiratory rate and a shortened expiratory time.^74^,^75^ Under acute hypoxic exposure (such as a high-altitude environment), hyperventilation mediated by the carotid body leads to respiratory alkalosis. The elevated plasma pH inhibits the activity of RTN neurons, significantly diminishing their responsiveness to afferent signals from the carotid body.^76^ This pH balance-based negative feedback regulation mechanism not only limits the intensity of the HVR but may also constitute an important pathological basis for the development of high-altitude illnesses. In contrast, when the normal tonic input from the carotid body decreases (e.g., due to hyperoxia), Phox2b^+^ neurons in RTN compensatory enhance respiratory drive by increasing CO_2_ sensitivity to maintain ventilation stability.^76^ Moreover, astrocytes in the RTN also participate in respiratory regulation through a unique oxygen-sensing mechanism. Specifically, astrocytes in the RTN can express transient receptor potential channel A1 (TRPA1). TRPA1 acts as a key oxygen sensor and can rapidly initiate compensatory responses under moderate acute hypoxic stimulation.^77^,^78^ In normoxic conditions, oxygen-dependent proline hydroxylation activates the E3 ubiquitin ligase, neural precursor cell-expressed developmentally down-regulated protein 4 (NEDD4-1). NEDD4-1 continuously mediates the ubiquitin-mediated degradation of TRPA1 channel proteins, maintaining a baseline level of channel activity.^79^ However, when the partial pressure of oxygen decreases, degradation of TRPA1 proteins is inhibited. This leads to an increase in the membrane surface density of the channels, triggering Ca^2+^ influx and adenosine triphosphate release from astrocytes, ultimately enhancing excitatory output from the respiratory center.^80^ Notably, functional studies indicate that the RTN primarily participates in CO_2_-induced arousal response regulation but shows a relatively limited response to pure hypoxic stimulation.^81^,^82^ To sum up, these studies underscore the important role of the RTN as a central carbon dioxide sensor in pH-dependent feedback regulation.

Long-term exposure to hypoxia can induce neuroplastic changes in the RTN. A study has shown a progressive decrease in the density of hypoxia-sensitive neurons projecting from the NTS to the RTN in Parkinson’s disease models.^64^ This adaptive change may lower the respiratory center’s threshold for responding to peripheral chemical signals, and thus prevent acid-base imbalances and metabolic disturbances caused by sustained hyperventilation. Interestingly, pathological analyses of some cases of sudden infant death syndrome have revealed structural defects or functional abnormalities in the RTN and the serotonergic neurons of the brainstem involved in their regulatory network.^83^,^84^ Animal experiments have confirmed that mice with specific loss of serotonergic neurons exhibit extremely high mortality rates during developmental stages. Their characteristic phenotype includes severe neonatal apneas and a complete loss of CO_2_-induced arousal responses. Further studies discovered that lesion models of the raphe nuclei in neonatal rodents can significantly weaken serotonergic input signals, and result in a marked reduction in hypoxia-triggered compensatory gasping reflexes.^85^ Therefore, in recent years, scholars have proposed that RTN neurons may act as relays and amplifiers for serotonergic neurons and other chemoreceptors in response to pH changes, rather than serving as pH sensors themselves.^86^ Thus, the functional coupling between the serotonergic system and RTN plays an irreplaceable role in maintaining the stability of basic respiratory rhythms, but the specific mechanisms involved still require further investigation.

In summary, the RTN serves as a central hub for chemosensation. It integrates inputs from peripheral chemoreceptors, glial cell signals, and various neurotransmitters to play a crucial role in maintaining respiratory homeostasis. Dysregulation of these mechanisms may be associated with many pathological conditions, such as sleep apnea and sudden infant death syndrome. Future research will continue to explore the network connections between the RTN and other respiratory and sympathetic centers.

## Rostral Ventral Lateral Medulla and Hypoxia

The RVLM contains two important functional units: the C1 catecholaminergic neuron group that regulates sympathetic nerve excitability and hypoxic responsiveness, and the central chemoreceptive area that partially overlaps with RTN. RVLM is a critical brain region for regulating sympathetic activity. It generates and maintains the tone of sympathoadrenal and sympathetic vasomotor nerves.^87^ C1 neurons express the enzymes necessary for norepinephrine synthesis. They send excitatory impulses and project to the lateral column of the spinal cord’s intermediolateral zone to activate sympathetic activity.^88^ When RVLM neurons are excited, they increase sympathetic nerve activity and raise blood pressure. Conversely, when neurons in the caudal ventral lateral medulla are activated, they inhibit the activity of RVLM neurons.^89^ Additionally, RVLM regulates multiple systems by integrating respiratory rhythm generation,^87^ maintaining glucose homeostasis,^90^ participating in inflammatory responses,^91^ and regulating alertness.^92^ It can also sense the internal environment and respond specifically to tissue acidification, hypoxia, and changes in intracranial pressure.^93^

### C1 neurons: key neurons in the rostral ventral lateral medulla involved in the hypoxia response

C1 neurons are not only key functional neurons within the core cardiovascular regulatory center, RVLM, but also hypoxia-sensitive neurons. Under physiological conditions, the baseline low firing activity of C1 neurons in the RVLM maintains sympathetic nerve tone to ensure blood pressure homeostasis.^94^ When stimulated by hypoxia or hypercapnia, C1 neurons can reshape their firing frequency in response to chemical stimuli via the reduced activity of inhibitory neurotransmitters (glycinergic and GABAergic). This electrical activity remodeling is considered a critical basis for the activation of the central chemosensitivity in vascular tone regulation.^95^ Indeed, hypoxia exerts a dual regulatory effect on the excitability of vasomotor neurons. On one hand, it relies on the indirect activation of neuronal excitability through afferent signals from the carotid body. For instance, during hypoxic exposure, carotid body afferent signals strongly drive C1 neurons, enhancing sympathetic output. This, in turn, triggers a pressor response and optimizes peripheral blood oxygen distribution.94 On the other hand, hypoxia directly modulates neuronal excitability through intrinsic cellular mechanisms within C1 neurons.^96^ Therefore, under hypoxic stimulation, C1 neurons play a key role in controlling sympathetic nervous tone. Additionally, studies have shown that moderate hypoxic stimulation can trigger the release of neuroendocrine factors, such as vasopressin, OT and adrenocorticotropic hormone.^97^,^98^ These responses are partially mediated by C1 neurons, and then achieve a synergistic interaction between autonomic regulation and neuroendocrine function.^88^ Consequently, C1 neurons in the RVLM region are not only pivotal nodes for sympathetic activation during hypoxic stress but also multifunctional hubs that integrate peripheral chemical signals, central electrical activity remodeling, and endocrine cascades.

### Role of rostral ventral lateral medulla in acute hypoxia

A recent study has shown that acute hypoxic stimulation can activate C1 neurons in the RVLM. These C1 neurons then enhance their neural connections with the PVN through glutamatergic projections.^99^ Burke et al.^18^ discovered that C1 neurons exhibit significant stage-specific regulation of sleep-wake transitions. During non-rapid eye movement sleep, activation of C1 neurons induces awakening by increasing respiratory rate and blood pressure, as well as triggering a sigh response. In contrast, during rapid eye movement sleep, activation of the same neurons only elevates blood pressure without affecting respiratory regulation. Thus, sighs or awakenings cannot be triggered.^18^ Interestingly, optogenetic stimulation of C1 neurons during wakefulness synchronously enhances blood pressure, respiratory frequency, and sighing responses. This suggests that their functional state is dynamically regulated by the level of consciousness.^18^ However, it remains to be further investigated whether C1 neurons mediate these effects through specific neural circuits, such as the locus coeruleus or the basal forebrain, which are critical centers for arousal.

### Role of rostral ventral lateral medulla in chronic hypoxia

RVLM plays a crucial role in regulating sympathetic activity and should not be overlooked in the development of cardiovascular diseases caused by chronic hypoxia. A previous study has found that CIH can reshape the sensitivity of RVLM neurons to adrenomedullin, enabling it to exert excitatory neural regulation under hypoxic conditions.^100^ Recent research has demonstrated that hypoxia significantly promotes the release of adrenomedullin, which is closely related to the abnormal enhancement of chemical sensory inputs in the NTS. This abnormal enhancement projects to the RVLM and drives the excessive activation of sympathetic excitatory network. As a result, it induces autonomic and cardiovascular dysfunction during CIH.^101^ Meanwhile, studies have indicated that the activation of NOD-like receptor family pyrin domain-containing 3 (NLRP3) inflammasome in the RVLM correlates with an enhanced HVR after hypoxia. A gene knockout experiment demonstrates that NLRP3 knockout significantly attenuates the CIH-induced enhancement of HVR in mice.^102^ Additionally, hypoxia can induce RVLM neurons to synthesize erythropoietin. This endogenous neuromodulator may contribute to hypertension by regulating central sympathetic output.^103^ These findings collectively reveal that the RVLM shapes the pathophysiological effects of CIH through multiple mechanisms, including neural signal transmission, inflammation pathway activation, and the release of endogenous neuromodulators.

Overall, the C1 neurons in the RVLM region play a critical role in the hypoxic response. They maintain the stability of vital signs by regulating mechanisms, such as respiratory reflexes, vasoconstriction, and sleep-wake states. These findings enhance our understanding of how the central nervous system integrates peripheral chemosensory signals to regulate autonomic and endocrine functions, and this regulation is essential for maintaining vital signs and responding to physiological stresses like hypoxia. Future research could further explore the role of C1 neurons in other physiological and pathological processes. Additionally, investigating regulatory approaches targeting these neurons may reveal their potential for clinical applications, providing new strategies for the prevention and treatment of related diseases.

## Parabrachial Nucleus and Hypoxia

The PB is a cluster of neurons located in the lateral reticular structure of the pontine tegmentum, surrounding the superior cerebellar peduncle. Functionally, it can be divided into two main parts: the medial PB and the LPB.^104^ PB contains various types of neurons. These neurons can express neuroactive substances such as calcitonin gene-related peptide, cholecystokinin, and somatostatin. This suggests that different types of neurons may participate in various regulatory functions through distinct neural projections. As an important sensory relay nucleus, PB can transmit sensory information from the brainstem to multiple regions in the forebrain, while also receiving afferent feedback from these brain areas. PB receives convergent inputs from RTN neurons,^70^ caudal NTS neurons relaying carotid body inputs,^105^ serotonergic neurons of the raphe nuclei,^106^ and C1 neurons of RVLM.^18^ Meanwhile, PB neuronal axons project to regions such as the thalamus,^107^ central amygdala,^108^ and bed nucleus of the stria terminalis.^109^ These projections contribute to modulating arousal, emotional processing and sensory integration.

### The main function of parabrachial nucleus in hypercapnic hypoxia

In recent years, the role of PB in hypercapnic hypoxia has gradually been recognized. Kaur et al.^110^ indicated that PB serves as the central hub mediating the arousal reflex induced by hypercapnia. Activation of glutamatergic neurons in the LPB is directly related to the arousal response during sleep caused by hypercapnia.^110^ Further studies have demonstrated that specific subgroups of LPB glutamatergic neurons, such as calcitonin gene-related peptide-positive neurons, act as molecular switches for CO_2_-driven arousal. Inhibition of calcitonin gene-related peptide-positive neurons completely blocks the arousal effects of hypercapnia without affecting CO_2_-induced respiratory compensation. This separation suggests distinct pathways for arousal and respiratory regulation.^111^ In contrast, the Phox2b^+^ glutamatergic neurons at the border of the Kölliker–Fuse nucleus and LPB specifically regulate the increase in respiratory frequency driven by CO_2_, with no direct association with the arousal response. However, medial PB does not exhibit functional involvement in this process.^112^ Therefore, the PB achieves functional decoupling of respiratory regulation and the arousal reflex through its specialized subregions.

### Role of parabrachial nucleus in acute hypoxia

In addition to its critical role in the arousal response to hypercapnia, animal studies have shown that acute hypoxemia can enhance post-inspiratory firing activity of the vagus nerve by activating the Kölliker-Fuse/PB complex circuit. This timing-specific regulation can optimize the efficiency of lung ventilation/perfusion matching and improve the body’s ability to uptake oxygen.^113^

In addition, neurons activated by hypoxia may relay signals through the NTS to the Kölliker-Fuse/PB complex. This pathway likely plays a key role in cardiopulmonary regulation.^114^ However, current research on hypoxia-related circuits involving the PB remains limited. Most studies focus on its ascending and descending projections related to pain,^115^,^116^ emotion,^117^ and feeding.^118^

Although the role of PB in hypoxia has been partially elucidated, the specific mechanism of PB in the hypoxia response still requires in-depth exploration. In the future, this field urgently needs to break away from the traditional hypercapnic hypoxia research framework and reveal the core functions of the PB in hypoxia adaptation from multiple dimensions, in order to provide new targets for respiratory failure, high-altitude diseases, and improvement of hypoxia tolerance.

## Paraventricular Nucleus of the Hypothalamus and Hypoxia

The PVN is located in the hypothalamic supraoptic region, on both sides of the third ventricle. It serves as an important integrative center for neuroendocrine and autonomic activities and plays a crucial role in cardiovascular regulation. PVN contains peptidergic neurons that secrete peptide hormones and comprises both magnocellular and parvocellular divisions.^119^ Among these, the axons of the magnocellular neurons primarily project to the neurohypophysis, releasing OT and arginine vasopressin. These hormones regulate fluid balance, uterine contractions, and stress responses.^120^ In contrast, the parvocellular neurons mainly secrete thyrotropin-releasing hormone and CRH to the anterior pituitary. These hormones initiate the hypothalamic-pituitary-thyroid axis and the hypothalamic-pituitary-adrenal axis.^120^ PVN is a higher-level regulatory center for visceral activities. Notably, oxytocinergic neurons in PVN directly project to RVLM and the intermediolateral column of the spinal cord, regulating the activity of sympathetic preganglionic neurons.^121^ PVN also plays a significant role in reflex responses to hypoxia, and contributes to heart rate reduction, blood pressure elevation, and increased phrenic nerve activity.^25^ Furthermore, PVN is interconnected with the NTS,^24^ and is essential for autonomic control of arterial pressure and the reflex response to transient hypoxia.

### Role of paraventricular nucleus in acute hypoxia

PVN plays a crucial role in acute hypoxia regulation by integrating brainstem projections and neuroendocrine signals. PVN receives dense projections from catecholaminergic neurons in the RVLM.^122^,^123^ These neurons can be categorized into three distinct subtypes: C1, C2, and C3.^124^ However, only the C1 subtype mediates the acute hypoxic response. During acute hypoxia, C1 neurons excite the small cell neurons in the PVN through glutamatergic projections.^99^ They also lower the threshold of the PVN’s response to hypoxia via catecholaminergic inputs.^88^ Moreover, PVN forms a negative feedback loop with glutamatergic projections from NTS, preventing excessive sympathetic activation. This feedback is essential for complete cardiopulmonary reflexes in response to hypoxia.^43^ In addition, acute hypoxia activates OT-positive neurons and CRH-positive neurons in the PVN.^125^ This activation promotes the release of OT and CRH, thereby mediating the endocrine system’s physiological adjustments in response to acute hypoxia.^126^ Hence, as a core node integrating hypoxic stress, PVN regulates sympathetic and endocrine changes during hypoxia through specific inputs from C1 neurons, NTS feedback, and the release of OT and CRH.

### Role of paraventricular nucleus in chronic hypoxia

PVN is involved in coordinating the balance between the autonomic nervous system and the endocrine system during chronic hypoxia. First, the long-term potentiation effect (long-term facilitation) of neuroplasticity produced by PVN after intermittent hypoxia is a crucial basis for its involvement in chronic compensatory regulation.^127^ Mechanistically, OT-positive neurons in the PVN release glutamate and OT in a coordinated manner. This specific activation impacts the heart’s parasympathetic neurons in the dorsal motor nucleus of the vagus and the nucleus ambiguus.^128^ In CIH, this pathway effectively counteracts excessive sympathetic nervous system activation, improving hypoxic hypertension.^10^ Chronic hypoxia significantly increases right ventricular systolic pressure in mice. In contrast, overexpression of angiotensin-converting enzyme 2 in CRH neurons of PVN can exert a protective effect against hypoxia-induced pulmonary hypertension.^129^ Additionally, β-endorphins can participate in the negative regulation of thyrotropin-releasing hormone release during hypoxic stress by inhibiting the release of thyrotropin-releasing hormone from the median eminence and PVN.^130^ However, selective destruction of catecholaminergic neurons projecting from the PVN to the brainstem (e.g., using anti-dopamine β-hydroxylase–saporin) can lead to reduced baseline respiratory rate, weakened HVR and impaired ability to maintain oxygen homeostasis. This further confirms the central role of PV in the integration of respiratory and cardiovascular functions.^131^ Furthermore, it has been reported that male offspring, but not females, born after gestational intermittent hypoxia exposure show increased c-Fos labeling density in the PVN. This indicates a persistent and sex-specific impact of gestational intermittent hypoxia on offspring health.^132^ Thus, the multi-layered regulatory network of PVN in hypoxic response provides dual neuroendocrine safeguards for maintaining homeostasis.

In summary, PVN is a key region in the hypoxic response. It dynamically balances acute compensation and chronic adaptation through multiple mechanisms, and serves as a critical node in the pathophysiological transformation related to hypoxia.

## Limitations

This review has several limitations. First, due to the breadth of hypoxia-related central nervous system research, this review may not cover all relevant literature. Second, while this review focuses on neural circuits and molecular changes in specific central nuclei, the detailed mechanisms of neurotransmitter interactions and downstream signaling cascades were not comprehensively discussed. Third, most studies are conducted in rodent models, and the translatability of these findings to human pathophysiology remains to be validated.

## Conclusion

**Figure 4:**
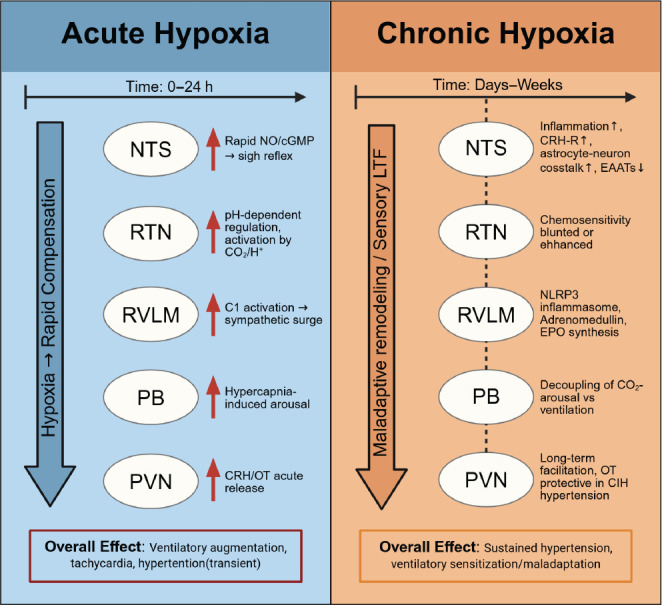
Differential mechanisms of acute versus chronic hypoxia-induced plasticity in central nuclei. Acute hypoxia (0–24 hours) triggers rapid compensatory responses, including ventilatory augmentation and transient sympathetic activation. Chronic hypoxia (days–weeks) induces maladaptive remodeling with sustained hypertension and ventilatory sensitization. Created with BioRender.com. cGMP: Cyclic guanosine monophosphate; CIH: chronic intermittent hypoxia; CO_2_: carbon dioxide; CRH: corticotropin-releasing hormone; EAATs: excitatory amino acid transporters; EPO: erythropoietin; NLRP3: NOD-like receptor family pyrin domain-containing 3; NO: nitric oxide; NTS: the nucleus of the solitary tract; OT: oxytocin; PB: parabrachial nucleus; PVN: paraventricular nucleus of hypothalamus; RTN: retrotrapezoid nucleus; RVLM: rostral ventral lateral medulla.

**Figure 5:**
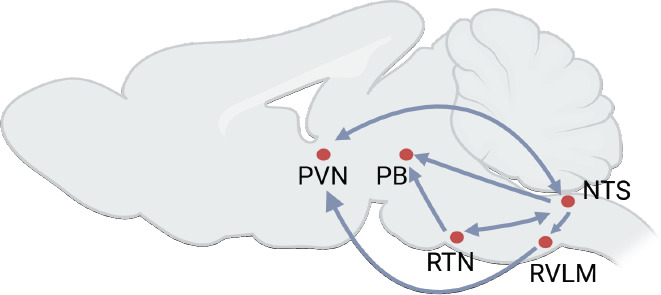
Neural circuit connectivity in hypoxia regulation. Created with BioRender.com. NTS: The nucleus of the solitary tract; PB: parabrachial nucleus; PVN: paraventricular nucleus of hypothalamus; RTN: retrotrapezoid nucleus; RVLM: rostral ventral lateral medulla.

Hypoxia is a pathological condition often coexisting with hypertension, obesity, metabolic disorders, and more. For example, patients with OSA experience recurrent intermittent hypoxia. This experience leads to a sustained state of high sympathetic nervous system activity, and gradually contributes to cardiovascular disorders. Therefore, addressing hypoxia itself, rather than merely treating complications like hypertension, is crucial for improving patient outcomes. In this review, we summarize five central nervous system mechanism regions associated with acute and chronic hypoxia (**Figure 4**). These regions are closely interconnected (**Figure 5**). Through various neurons, neurotransmitters, and glial cells, they jointly perform functions such as cardiopulmonary regulation under normoxia, hypocapnic hypoxia, and hypercapnic hypoxia conditions (**Table 1**). Based on specific physiological needs, these regions can adjust cardiopulmonary responses in a timely manner through afferent signals from carotid body chemoreceptors, helping to maintain homeostasis.

**Table 1:** Studies of the regulation of the central nervous system in response to changes in peripheral systems during hypoxia

	Relevant system	Hypoxia duration	Major mediated neuron types	Outcome	Reference
NTS	Breathing-related	Acute hypoxia	Grp^+^	Sighing	17
		Chronic hypoxia	VGlut2^+^	HVR	38 43 49 50
	Blood pressure–related	Chronic hypoxia	VGlut2^+^	Hypertension	44
	Endocrine-related	Chronic hypoxia	VGlut2^+^	OT, CRH↑	44 63
RTN	Breathing-related	Acute hypoxia	Phox2b^+^	HVR	76 77 78
		Chronic hypoxia	Phox2b^+^	HVR	83 84 85
RVLM	Breathing-related	Chronic hypoxia	C1	HVR	102
	Blood pressure–related	Chronic hypoxia	C1	Hypertension	100 101 103
	Endocrine-related	Chronic hypoxia	C1	VP, OT, ACTH, EPO↑	88 103
PB	Breathing-related	Hypercapnia	Phox2b^+^	Respiration rate↑	112
PVN	Endocrine-related	Acute hypoxia	OT^+^, CRH^+^	OT, CRH↑	125 126
	Breathing-related	Chronic hypoxia	Catecholaminergic	HVR	131
	Blood pressure–related	Acute hypoxia	Catecholaminergic	Sympathetic tone regulation	88
		Chronic hypoxia	VGlut2^+^, OT^+^	Hypertension	10 128
			CRH^+^	Hypertension	129

ACTH: Adrenocorticotropic hormone; CRH: corticotropin-releasing hormone; EPO: erythropoietin; Grp: gastrin-releasing peptide; HVR: hypoxic ventilatory response; NTS: nucleus of the solitary tract; OT: oxytocin; PB: parabrachial nucleus; PVN: paraventricular nucleus of hypothalamus; RTN: retrotrapezoid nucleus; RVLM: rostral ventral lateral medulla; VP: vasopressin.

**Figure 6:**
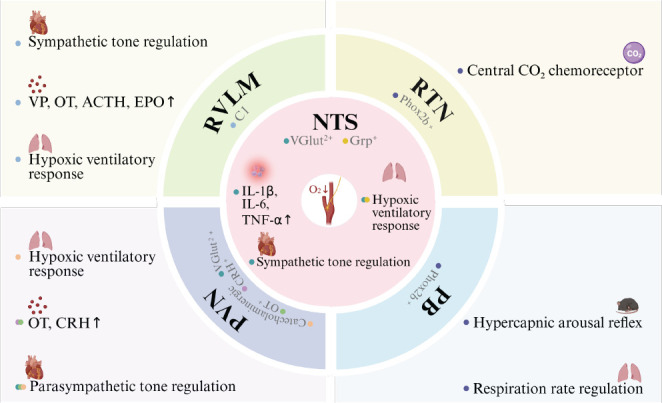
Central transmission mechanisms of hypoxia. Created with BioRender.com. ACTH: Adrenocorticotropic hormone; CRH: corticotropin-releasing hormone; EPO: erythropoietin; Grp: gastrin-releasing peptide; NTS: nucleus of the solitary tract; OT: oxytocin; PB: parabrachial nucleus; Phox2b: paired-like homeobox 2b; PVN: paraventricular nucleus of hypothalamus; RTN: retrotrapezoid nucleus; RVLM: rostral ventral lateral medulla; VGlut2: vesicular glutamate transporter 2; VP: vasopressin.

Current research on hypoxia-related neural mechanisms mainly focuses on core brain regions controlling respiration. Special attention is given to hypoxia-induced sensitization of sympathetic centers and regulation of cardiopulmonary reflex pathways. The NTS serves as a critical primary hub integrating peripheral hypoxic signals. The dynamic response and regulation of the HIF signaling pathway within the NTS are key to understanding hypoxia-adaptive neural remodeling (**Figure 6**). However, how HIF-mediated transcriptional regulation drives synaptic plasticity and neural circuit reorganization in the central nervous system, especially within cardiorespiratory integration centers, has not been systematically clarified. In addition, significant gaps remain in understanding the multisynaptic neural circuits underlying hypoxia-triggered cardiopulmonary reflexes. Although some studies show that projections from the NTS to the PVN are activated by hypoxia, cross-regional cooperative mechanisms involving the NTS, PB, hypothalamus, and amygdala require deeper investigation. Future research should address the following key directions: (1) Hypoxia neurobiology must transcend the single nucleus perspective. It should build a central regulatory framework based on dynamic integration of hierarchical multi-nucleus networks. This involves elucidating the cascade of hypoxic signals transmitted from peripheral chemoreceptors (e.g., carotid body) through primary integrative nuclei (NTS) to higher centers (PB, PVN, and amygdala). The temporal activation patterns of different nuclei during HVR and chronic metabolic adaptation should be clarified. Special attention should be paid to mechanisms of competition and cooperation among key nuclei. For example, the antagonistic balance between PB-mediated respiratory inhibition and rhythm generation by the pre-Bötzinger complex. Also, the fine coordination of sympathetic excitation and parasympathetic compensation (via the dorsal motor nucleus of the vagus) along hypothalamus-brainstem pathways. These interactions promote dynamic neuro-autonomic balance. (2) Clarify differences in neuroplastic remodeling and molecular markers at critical nodes (e.g., NTS, carotid body-central interfaces) between acute hypoxic stress and chronic hypoxic adaptation. (3) Explore whether these mechanisms can provide novel neuromodulation targets for treating comorbidities such as anxiety and depression secondary to OSA, and sympathetic hyperactivity in hypertension. This would promote clinical translation for hypoxia-related diseases. (4) Utilize high-field functional magnetic resonance imaging for whole-brain network analyses, genetically encoded lactate sensing probes for real-time monitoring, and combine fiber photometry with brain tissue oxygen partial pressure sensors. This will enable multiscale dynamic dissection of hypoxic neural responses. By deeply integrating circuit mechanism analysis, molecular dynamic tracing, and clinical phenotype correlation, this approach will systematically reveal general principles of neural adaptation to hypoxia. It will lay a theoretical foundation for precise neuromodulatory therapies in diseases, such as OSA, high-altitude illness, and heart failure complicated by hypoxia.

## Data Availability

*Not applicable.*
